# The influence of night-time bracing on curve progression is not affected by curve magnitude in adolescent idiopathic scoliosis: a study of 299 patients

**DOI:** 10.2340/17453674.2024.39965

**Published:** 2024-02-12

**Authors:** Martin HEEGAARD, Niklas TØNDEVOLD, Benny DAHL, Thomas B ANDERSEN, Martin GEHRCHEN, Søren OHRT-NISSEN

**Affiliations:** Spine Unit, Department of Orthopedic Surgery, Rigshospitalet, Copenhagen University Hospital, Copenhagen, Denmark

## Abstract

**Background and purpose:**

The efficacy of bracing larger curves in adolescent idiopathic scoliosis (AIS) patients is uncertain. We aimed to assess the influence of night-time bracing in AIS patients with main curves exceeding 40° Cobb angle at brace initiation.

**Methods:**

We reviewed AIS patients treated with nighttime braces between 2005 and 2018. Patients with curves ≥ 25° and estimated growth potential were included. Patients were monitored with radiographs from brace initiation until brace weaning at skeletal maturity. Patients were grouped based on curve magnitude at initial evaluation: a control group (25–39°) and a large-curves group (≥ 40°). Progression was defined as > 5° increase.

**Results:**

We included 299 patients (control group, n = 125; large-curves group, n = 174). In the control group, 65 (52%) patients progressed compared with 101 (58%) in the large-curves group (P = 0.3). The lower-end vertebra (LEV) shifted distally post-bracing in 41 (23%) patients in the largecurves group. Patients with progressive large curves were younger (age 13.2 [SD 1.5] vs. 13.9 [SD 1.1], P = 0.009) and more premenarchal (n = 36 [42%] vs. n = 6 [9%], P < 0.001) compared with non-progressive large curves.

**Conclusion:**

Progression risk in patients with curves exceeding 40° treated with night-time bracing is similar to smaller curves. The LEV moved distally in almost one-fourth of the larger curves, possibly affecting fusion levels in cases of surgery.

Bracing is the preferred nonoperative treatment for adolescent idiopathic scoliosis (AIS) patients with main curves ranging from 25° to 40° [1], while curves exceeding 45–50° are generally considered candidates for corrective surgery [2]. Curves exceeding 40° in the skeletally immature AIS patient pose a clinical dilemma considering the unknown efficacy of bracing in larger curves and the risk of carrying out fusion surgery in a growing child. Surgical treatment aims to both correct the spine and prevent further curve progression, therefore determining the optimal timing for surgery is crucial [2]. Delaying surgery until adulthood can be problematic due to increased curve rigidity requiring more aggressive correction techniques, resulting in higher complication rates [3]. Likewise, some studies even suggest that select AIS patients with open triradiate cartilage could benefit from final fusion to the stable vertebrae or spinal tethering [4-6]. Conversely, adolescent patients are generally advised to achieve skeletal maturity before undergoing fusion surgery to minimize the risk of crankshaft phenomenon, distal adding-on, or decompensation [7-9]. For skeletally immature AIS patients, bracing can be used as either final treatment or bridging prior to surgery. The purpose of bracing is to halt curve progression and thereby diminish the risk of surgery until skeletal maturity [10]. Limited literature is available regarding the efficacy of bracing for larger curves. This study aims to assess the impact of night-time bracing on curves larger than 40° in AIS patients compared with a control group (curve 25–39°). We hypothesize non-inferiority in terms of bracing influence on large curves compared with small curves.

## Methods

### Subjects and radiographs

**Figure 1:**
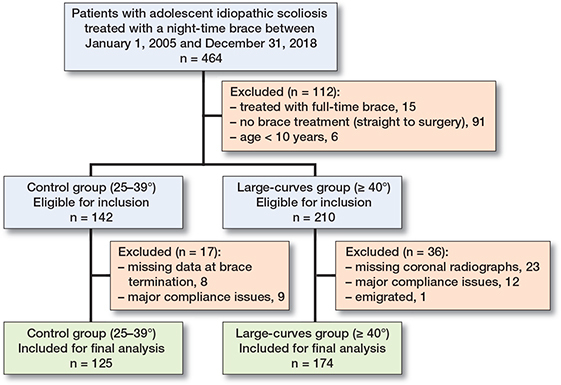
Flowchart of patient selection.

Patients with AIS treated with a night-time brace (Providence, Sahva, Copenhagen, Denmark) between January 1, 2005 and December 31, 2018, were all included. All patients with a main curve ≥ 25° and remaining growth based on either Risser stage (Risser stage < 3 or Risser stage 3–4 with signs of progression), Sanders stage (Sanders stage < 5 or Sanders stage 5–6 with signs of progression), or menarchal status (< 2 years post-menarche) were included. Non-compliance or missing anterior-posterior radiographs were exclusion criteria (Figure 1). The standard bracing regime for the night-time brace is a minimum 8 hours/day during sleep. Demographic data was collected using electronic medical records. On standing anteroposterior radiographs Cobb angle, Risser stage, and curve types were collected. Curve types were determined as either thoracic (apex > Th12), thoracolumbar/lumbar (apex ≤ Th12), or double curves with specified main curve (both curves > 30°). All included patients had radiographs taken before brace initiation and at brace termination with 1 night out-of-brace prior. Patients were followed until brace termination and surgical treatment was assessed 2 years after brace termination. Radiographs were analyzed using the validated software system KEOPS (SMAIO, Lyon, France) [11]. Brace treatment was discontinued at skeletal maturity defined as either 2 years post menarche, no height change between inhospital visits (6 months apart), or closed ulnar epiphyseal plate on wrist radiographs (Sanders stage 7) [1,12].

The present study adhered to the STROBE guidelines [13].

### Stratification of curve magnitude and progression

Patients were divided into 2 groups, depending on curve magnitude at brace initiation as suggested by SRS and SOSORT guidelines [1,14]: a control group (25–39°) and a large-curves group (≥ 40°). The large-curves group was stratified into 3 groups depending on Cobb angle magnitude: 40–49°, 50–59°, ≥ 60°, and furthermore into progressive and non-progressive curves. Radiographic progression was defined as Cobb angle increase of > 5° and non-progression as < 6° increase in the brace treatment period. The lower end vertebra (LEV) was defined as the most distal vertebra included in the main curve.

### Statistics

All statistical analysis was made using the software system R v. 4.2.2 (R Development Core Team, Vienna, Austria, 2020). We report data as either means (standard deviation [SD]), medians (interquartile range [IQR]), or counts (%). Histograms and Q–Q plots were used to assess data distribution. We used Student’s t-test and Wilcoxon’s rank-sum test according to data distribution. For categorical data, Pearson’s chi-square was used. To detect differences amongst 3 or more groups, we used one-way ANOVA and the Kruskal–Wallis test for normally- and non-normally distributed data, respectively. Logistic regression analysis was used to assess predictors of progression risk reported as odds ratios (OR) with 95% confidence interval (CI). Alpha levels of 5% were considered statistically significant.

### Ethics, registration, funding, and disclosures

Approval for the study was obtained from the National Health and Medical Authority and the National Data Protection Agency (May 20, 2020 #31-1521-327; Oct 21, 2021 #P-2021-779). The current study did not pre-register the protocol, and there was no receipt of funds. Authors report no conflict of interests directly related to this work. Complete disclosure of interest forms according to ICMJE are available on the article page, doi: 10.2340/17453674.2024.39965

## Results

**Figure 2:**
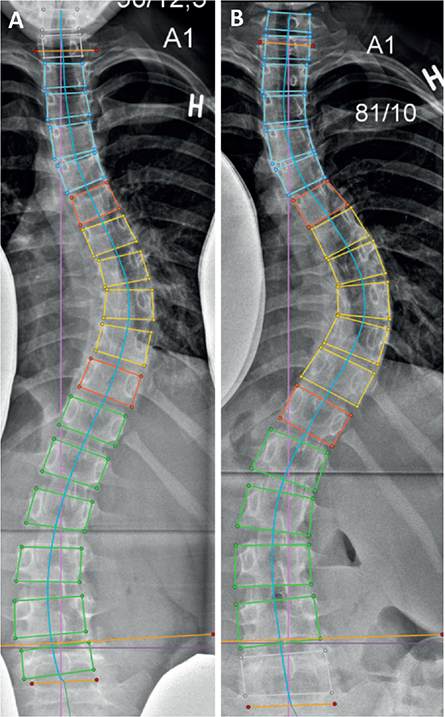
A premenarchal girl, aged 13, diagnosed with adolescent idiopathic scoliosis presenting a main curve of 45°. Upon reaching skeletal maturity, brace treatment was discontinued, and the main curve had progressed to 58°, with the lower end vertebra shifting 1 level distally. Subsequently, the patient underwent posterior instrumentation from Th4 to Th12.

We included 299 patients in the final analysis with 125 patients in the control group and 174 patients in the large-curves group (Figure 1). 7% (n = 21) of patients were excluded due to major compliance issues, specifically premature brace discontinuation. The 2 groups were similar in age (13.7, SD 1.3 vs. 13.5, SD 1.4, P = 0.2) and sex distribution (female 87% vs. male 93%, P = 0.1), but the large-curves group were less often premenarchal (46% vs. 28%, P = 0.004) (Table 1). Curve progression was observed in 52% of patients in the control group and 58% in the large-curves group (P = 0.3) (Table 1). Progression rate ranged from 58% for 40–49° curves to 62% in the ≥ 60° curves (P = 0.9) (Table 2). The surgical rate varied significantly depending on curve magnitude. In the control group (25–39°), 38% (n = 47) of patients underwent surgery, whereas in the large-curves group (≥ 40°), this percentage increased to 75% (n = 131) (P < 0.001) (Table 1). Stratifying the large-curves group (40–49°, 50–59°, ≥ 60°), surgical rate differed significantly: 63% (n = 60), 84% (n = 42), and 100% (n = 29), respectively (P < 0.001) (Table 2). Both the main curve and secondary curve increased post night-time bracing in the large-curves group (P < 0.001) (Table 3). The LEV moved ≥ 1 vertebra distally in 41 (23%) patients post bracing, corresponding to 37 (27%) thoracic main curves and 4 (11%) lumbar main curves (Table 3, Figure 2). Patients with progressive larger curves (≥ 40°) were younger (13.2 years vs. 13.8 years, P = 0.009) and more often premenarchal (42% vs. 9%, P < 0.001) compared with non-progressive larger curves. In multivariate logistic regression analysis, premenarche was statistically significantly associated with progression in the large-curves group ( OR 6, CI 2–17) (Table 4).

**Table 1:** Patient demographics in control group and large-curves group

	Control (25–39°) n = 125	Large curves ( ≥ 40° ) n = 174	P value
Pre-brace main curve Cobb angle, median (IQR), °	33 (30–36)	49 (43–55)	–
Patients with main curve progression, n (%)	65 (52)	101 (58)	0.3
Age, mean (SD)	13.7 (1.3)	13.5 (1.4)	0.2
Female, n (%)	109 (87)	162 (93)	0.1
Premenarchal, n (%)	48 (46)	42 (28)	0.004
Missing data	4	9	
Fusion performed at 2-year follow-up, n (%)	47 (38)	131 (75)	< 0.001

**Table 2:** Patient demographics for curves ≥ 40° stratified depending on curve magnitude at brace initiation

	Curve magnitude, Cobb angle			P value
	40–49° n = 95	50–59° n = 50	≥ 60°n = 29	
Pre-brace main curve Cobb angle, median (IQR), °	44 (42–46)	53 (51–55)	65 (62–74)	–
Patients with main curve progression, n (%)	55 (58)	28 (56)	18 (62)	0.9
Age, mean (SD)	13.5 (1.5)	13.5 (1.3)	13.3 (1.4)	0.7
Female, n (%)	88 (93)	47 (94)	27 (93)	1.0
Premenarchal, n (%)	24 (29)	14 (32)	4 (15)	0.3
Missing data	5	3	1	
Fusion performed at 2-year follow-up, n (%)	60 (63)	42 (84)	29 (100)	< 0.001

**Table 3:** Patient demographics and radiographic parameters for the largecurves group (≥ 40°; n =174) before and after brace treatment

	Pre-brace	Post-brace	P value
Age, mean (SD)	13.5 (1.4)	15.2 (1.5)	–
Premenarchal, n ^a^	42 (28)	0 (0)	< 0.001
Main curve Cobb angle, median (IQR), °	49 (43–55)	58 (49–69)	< 0.001
Global balance, median (IQR), mm	9 (3–20)	15 (5–27)	< 0.001
T1 inclination, median (IQR), °	3 (1–6)	4 (2–8)	< 0.001
Secondary curve Cobb angle, mean (SD), °	32 (9)	38 (13)	< 0.001
Lower end vertebra level, n			–
Unchanged	–	133	
+1	–	35	
+2	–	6	
Curve type, n (%)			0.02
Thoracic	73 (59)	39 (22)	
Thoracolumbar/lumbar	40 (32)	8 (5)	
Double curves			
Main thoracic	8 (6)	99 (57)	
Main thoracolumbar/lumbar	4 (3)	28 (16)	

a Missing data: n = 9.

**Table 4:** Logistic regression analysis on progression for the large-curves group

	Univariable OR (CI)	P value	Multivariable OR (CI)	P value
Pre-brace Cobb angle	1.0 (1.0–1.1)	0.6	1.0 (1.0–1.1)	0.3
Age	0.7 (0.6–0.9)	0.01	0.8 (0.6–1.1)	0.2
Premenarche	7.6 (3.0–19.5)	< 0.001	6.2 (2.3–17.0)	< 0.001
Curve type				
Double major	4.1 (1.6–10.8)	0.004	2.4 (0.8–7.2)	0.1
TL/L	1.5 (0.7–3.2)	0.3	1.2 (0.5–2.9)	0.7

OR = odds ratio; CI = 95% confidence interval; TL/L = thoracolumbar/lumbar.

## Discussion

The current study aimed to assess the influence of night-time bracing on skeletally immature AIS patients with main curves ≥ 40°. We found similar progression rates across curve magnitude but the surgical rate differed depending on the size of the curve. The main and secondary curve increased post bracing and the LEV changed to a more distal vertebra in almost one-fourth of the patients with large curves. Patients with progressive large curves were generally younger and more often premenarchal compared with non-progressive larger curves.

While the literature extensively covers the influence of bracing for curves < 40°, there is limited data concerning bracing in larger curves [15]. Our results demonstrate similar progression rates irrespective of curve magnitude, indicating that the influence of the night-time brace is not determined by the size of the curve. We observed similar progression rates in both our control group and large-curves groups compared with the existing literature [15-19]. Verhofste et al. demonstrated the efficacy of full-time bracing in postponing surgery until skeletal maturity in AIS patients with curves ≥ 40°, reporting a 42% success rate for nonoperative treatment and a 47% stabilization/improvement in the main curve [16]. Some studies on full-time bracing reported a notably lower progression rate (4%, 24%) on curves > 45° [20,21]. These cohorts included juvenile scoliosis with much longer brace wear (5 years), hindering direct comparison with our findings. Generalizing our data on the influence of night-time braces to encompass all brace treatments and scoliosis variations presents challenges and should be approached with caution. To our knowledge, a single study previously examined night-time bracing efficacy in AIS patients with curves exceeding 40° [22]. This study found a 23% progression risk for patients with curves ranging from 40–45°, though it had a limited sample size of 13 patients, all within 1 year of menarche [22]. To better represent clinical practice, we included patients with remaining growth, considering not only menarche status but also Risser and Sanders stages.

When assessing surgical rate, the general recommendation is to perform surgery in AIS patients with curves above 45/50° [2]. In our large-curves group (≥ 40°), nonoperative treatment proved successful in 25% of cases. Categorizing 50° and above as a direct indication for surgery, our study found a 16% nonoperative success rate in curves ranging from 50–59°. This implies that the brace is beneficial, even in cases where surgery is advised. All patients with baseline curves exceeding 60° underwent surgery, but the application of braces in these patients presents an opportunity to postpone surgery until skeletal maturity. Verhofste et al. reported similar 15% (n = 2) nonoperative treatment success for full-time braced patients with curves exceeding 50° [16]. The decision between opting for surgery and accepting the associated revision risk or initiating night-time bracing for approximately 2 years with LEV moving 1 level down is a clinically relevant discussion for the families. It is crucial to note that bracing may introduce additional mental stress, and curve progression may not only impact LEV but also influence pulmonary function [23,24].

Choosing the appropriate fusion levels in AIS patients has been a topic of ongoing debate among surgeons. Proposed landmarks for this decision include the LEV, stable vertebra, and neutral vertebra [4,8,25]. In our study, LEV moved distally in almost one-fourth of the patients post bracing, possibly influencing the number of fusion levels in cases of surgery. Clearly, defining lower level of fusion cannot be solely decided by LEV, and includes multiple factors such as curve type, flexibility, surgical technique, surgical approach, and instrumentation used.

Young age, premenarche, low Risser stage, open triradiate cartilage, decreased brace-wear time, and lack of initial inbrace correction among others are known predictors of curve progression [26,27]. It would seem logical to generalize predictive factors of progression from smaller curves to larger curves, but clear evidence is needed. We found patients with progressive curves above 40° to predominantly be younger and more often premenarchal compared with non-progressive curves. These findings are in alignment with previous studies assessing curves above and below 40° [16,28]. We did not investigate predictive factors such as Risser stages, in-brace correction, or brace wear time and therefore cannot draw any conclusions on this.

This study is not without limitations. Our control group consists of AIS patients with curves ranging from 25–39°, whereas a better fitted control group would be AIS patients with curves exceeding 40° and no brace treatment. As a result, we cannot distinguish between the impact of the brace and the natural progression of AIS patients over time. We also lack a comparative group of patients who underwent treatment with the full-time brace, preventing us from drawing conclusions regarding its influence on larger curves. Patients were enrolled over a long period, which could introduce changes in diagnostics and bracing criteria. Throughout the study period, the criteria for prescribing bracing have evolved. Initially, emphasis relied heavily on factors like Risser and Tanner stages as well as age and height gain. The current approach places greater importance on a combination of various more precise skeletal maturity factors, including, but not limited to, Sanders stages, menarche status, and olecranon ossification centers. We excluded 7% of patients because of major compliance issues (discontinuation of the brace prematurely), hence increasing selection bias. The night-time brace in our institution lacks heat sensors for compliance monitoring. Notably, patients either consistently wear the night-time brace throughout the night or do not wear it at all, with minimal instances of brace removal during the night. This characteristic diminishes the significance of hourly wear detection with heat sensors for the night-time brace [29]. Consequently, our compliance assessment relies on patient reports during in-hospital control visits, introducing potential inaccuracies that may impact the accuracy of our results.

### Conclusion

The influence of the night-time brace is not determined by curve magnitude. We found similar progression rates regardless of curve size in skeletally immature AIS patients, arguing that bracing is a viable treatment option either as definitive treatment or as bridging before surgery in all curves ≥ 25°. This is clinically relevant information in the dialogue with patients and their families. The LEV moved distally in almost one-fourth of patients, possibly affecting fusion levels in cases of surgery. Patients with progressive curves were younger and more often premenarchal, like patients with curves below the surgical threshold of 45–50°. Whether night-time bracing is a superior choice to observation for AIS patients with larger curves could be a focus for future studies.

## Notes

MH and SO: Contributed to the conception or design of the work, and to the acquisition, analysis and interpretation of data, and revised the work. BD, MG, NT, and TBA: Contributed to the conception or design of the work and revised the work.

Handling co-editor: Ilkka Helenius

*Acta* thanks James O Sanders and Stuart L Weinstein for help with peer review of this manuscript.
